# Depth Imaging Through Smoke Using Nonparametric Estimation for Array Gm-APD LiDAR

**DOI:** 10.3390/s26113330

**Published:** 2026-05-24

**Authors:** Yinbo Zhang, Qingyu Hou, Haoyan Wang, Boteng Zhang, Jialong Zhou, Jianfeng Sun

**Affiliations:** 1 National Key Laboratory of Laser Spatial Information, Institute of Opto-Electronic, Harbin Institute of Technology, Harbin 150001, China; zhangyinbo_hit@163.com (Y.Z.); whywhy200305@163.com (H.W.); 18901798253@163.com (B.Z.); m13962878908@163.com (J.Z.); 2 Research Center for Space Optical Engineering, Harbin Institute of Technology, Harbin 150001, China; houqingyu@126.com; 3 Zhengzhou Research Institute of Harbin Institute of Technology, Zhengzhou 450000, China

**Keywords:** array Gm-APD LiDAR, dynamic smoke occlusion, Pearson correlation, non-parametric estimation, depth imaging

## Abstract

Array Gm-APD LiDAR is highly vulnerable to strong backscattering caused by dynamic smoke. Conventional depth imaging methods cannot rapidly identify the smoke occlusion state, which greatly reduces the target recovery quality of the reconstructed depth image. To solve this problem, this paper presents a non-parametric algorithm for rapid smoke detection and depth imaging for array Gm-APD LiDAR. The proposed method does not rely on parameter estimation of the echo model. Instead, it determines the presence of smoke occlusion by calculating the Pearson correlation coefficient between the echo signal obtained from the superposition of all array pixels and the instrument response function. In this way, the method rapidly identifies smoke interference in a single depth image, performs fast denoising, and reconstructs the depth image. In a dynamic smoke environment with an average attenuation length of no more than 5.1, the proposed algorithm achieves 100% accuracy in occlusion discrimination based on 250 frames of array data. When the smoke occlusion rate reaches 96% and the average attenuation length is 2.29, the method obtains a target recovery of 0.71, which is 86.8% higher than that of the conventional algorithm. These results indicate that the proposed method has strong practical value for array Gm-APD LiDAR, especially for high-speed depth imaging in harsh atmospheric environments with severe obscuration.

## 1. Introduction

Array Gm-APD LiDAR offers single-photon sensitivity and direct planar imaging capability. It is widely used in three-dimensional imaging tasks, such as long-range 3D imaging [1,2], unmanned aerial vehicle (UAV) detection [3,4], and high-resolution ground mapping [5]. However, these applications often face interference from atmospheric obscurants, including smoke, rain, fog, and haze. In contrast to conventional infrared imaging techniques [6,7,8], Gm-APD LiDAR typically operates in the near-infrared band. However, when laser signals propagate through highly absorbing and scattering media, both transmission efficiency and imaging performance deteriorate significantly. Moreover, strong backscattering from such obscurants can induce a severe pile-up effect in Gm-APD LiDAR [9], thereby further degrading the accuracy of target detection and depth reconstruction. This effect significantly lowers the echo signal-to-noise ratio. In addition, the backscattered signal is highly similar to the target echo in the time domain, and its intensity is often stronger than that of the target signal. As a result, conventional reconstruction algorithms tend to treat the interference signal as target information, which greatly degrades the quality of the recovered depth image.

To overcome this problem, many studies focus on depth imaging through atmospheric obscurants using single-photon LiDAR. In 2019, Tobin [10] applied pixel-wise cross-correlation [11], RDI-TV [12], and UA [13] to reconstruct depth information in obscured scenes. In 2020, Halimi [14] proposed the multidimensional-nonlocal restoration of 3D (M-NR3D) algorithm. In 2021, Tobin [15] developed the median-based multi-scale restoration of 3D images (M2R3D) algorithm. In 2022, Shi [16] used a zero-order Bessel beam with nondiffractive characteristics as the illumination source in a single-photon LiDAR system. In 2023, Jiang [17] adopted an optical transceiver with an extremely narrow field of view and combined a 3D deconvolution method based on a convex optimization solver [18] with a nonlocal neural network [19]. This approach achieves depth imaging of a building at 13.4 km in an outdoor foggy environment. In 2025, Wang [20] developed two iterative reconstruction frameworks based on EM and gradient-based optimization, which effectively suppress fog-backscatter interference and achieve more accurate depth recovery. In 2025, Zhu [21] proposed a dual-branch tracking method (DBTM) for single-photon counting LiDAR target detection under water mist interference, with the positioning error of 0.161 m. In 2026, Huang [22] introduced vortex coherence filtering into a coaxial scanning single-photon LiDAR system for enhanced 3D imaging in dense fog, achieving a 37.56 times improvement in the raw signal-to-background ratio under visibility conditions as low as 0.61 m. These studies mainly improve imaging performance through optical system design and advanced reconstruction algorithms, thereby suppressing interference from atmospheric obscurants.

Another important research direction is depth imaging based on model parameter estimation. These methods separate signal photons from noise photons through pixel-wise estimation of echo models. In 2018, Satat [23] showed that fog echoes and target echoes can be described by Gamma and Gaussian distributions, respectively. Based on the Gamma model, Liu [24] proposed a single-parameter fog-removal imaging algorithm for single-photon LiDAR. In 2025, Peng [25] presented a fog-removal depth-imaging method that combines Gamma modeling with density-clustering-guided Gaussian fitting. Some researchers also established smoke echo models beyond the Gamma distribution. In 2020, Mau [26,27] fitted the echo signal with a mixed model of log-normal and Gaussian distributions. The expectation-maximization algorithm is then used for pixel-wise estimation in a 32 × 32 array SPAD LiDAR system. Sang [28] proposed a fog-removal method for fog-profile estimation based on a physical LiDAR model. In 2022, Laurenzis [29] reconstructed scene information by using the transient projection of a virtual Huygens–Fresnel wavefront. In recent years, our group also carried out depth imaging through indoor artificial smoke and outdoor fog by using model parameter estimation methods [30,31].

Existing studies show that atmospheric obscurants strongly affect the imaging process. In general, the detected echo contains both target-reflected photons and backscattered photons from the obscurants. However, under dynamic atmospheric conditions, obscurants do not always cause continuous blocking. Instead, they often appear only within limited spatial and temporal regions. Most traditional parameter estimation methods rely on complicated parametric models. This increases computational cost and reduces adaptability to changing environments. Therefore, these methods often perform poorly under dynamic smoke interference. In addition, model-based methods have limited ability to distinguish different signal types. They may incorrectly classify target echoes as smoke-scattering signals. Even in scenes without interference, they still increase computational burden and may reduce depth reconstruction quality. Therefore, efficient methods for rapid detection of atmospheric obscurants are still needed.

To address these issues, this paper proposes a non-parametric estimation algorithm for rapid smoke detection and depth imaging with array Gm-APD LiDAR. The proposed method consists of two main steps: signal-level non-parametric estimation for rapid smoke detection and image-level fusion of multi-frame depth images. This strategy avoids the complex pixel-wise model estimation required by conventional methods when processing array data. It rapidly determines whether smoke interference exists in a single depth image and then performs fast denoising and depth reconstruction. Therefore, the proposed method is suitable for depth imaging with array Gm-APD LiDAR in dense and dynamic atmospheric obscurant environments. It is expected to provide better performance than traditional parameter estimation methods under dynamic smoke conditions.

## 2. Materials and Methods

### 2.1. Model of Data

In the presence of atmospheric obscurants, the photons detected by a single pixel of an array Gm-APD LiDAR consist of target-reflected photons *N_t_*, backscattered photons *N_f_*, ambient background photons *N_b_*, and system noise photons *N_d_*. The time-domain expression of the received echo signal *S*(*t*) under atmospheric obscurant conditions is written as follows:

(1) $$S(t)=\eta N_{f}(t)+\eta N_{t}(t)+\eta N_{b}+N_{d}(t)$$

where *η* denotes the detector efficiency. In array Gm-APD LiDAR, the photon counts recorded in each pixel follow a Poisson distribution *P*(.), which can be expressed as

(2) $$H_{i}∼P\left(S_{i}\right)=\left\{\prod_{j=1}^{i-1}\exp\left(-S_{j}\right)\right\}\left\{1-\exp\left(-S_{i}\right)\right\}$$

where *H_i_* denotes the observed histogram at the *i*-th time bin. By taking the likelihood function of the above expression, the photon counts in the *i*-th time bin is obtained as follows:

(3) $$S_{i}=\ln\left(1+\frac{H_{i}}{N-\sum_{k=1}^{i}H_{k}}\right)$$

where *N* represents the total number of emitted laser pulses. The detailed derivation is provided in Formula (A1) in Appendix A.

For echo modeling, a Gaussian model is used to describe the target echo, while a Gamma model [31] is used to characterize the smoke echo. When the shape parameter *K* of the Gamma distribution is sufficiently large, the Gamma distribution approaches a Gaussian distribution. Based on this property, a double-Gamma distribution model is adopted to represent the overall echo signal. The corresponding expression is given as follows:

(4) $$S(t)=\eta \delta_{f}\frac{{\beta_{f}}^{K_{f}+1}}{\Gamma (K_{f})}t^{K_{f}}\exp(-\beta_{f}t)+\eta \delta_{t}\frac{\beta_{t}^{K_{t}+1}}{\Gamma (K_{t})}t^{K_{t}}\exp(-\beta_{t}t)+\eta N_{b}+N_{d}(t)$$

where *β_f_* is the reciprocal of the scale parameter of the smoke echo signal. *K_f_* is the shape parameter of the smoke echo signal. Γ(*•*) is the Gamma function. *δ_f_* is the intensity value of the smoke echo signal. *β_t_* is the reciprocal of the scale parameter of the target echo signal. *K_t_* is the shape parameter of the target echo signal. *δ_t_* is the intensity value of the target echo signal. The detailed derivation of this model is presented in Appendix B.

### 2.2. Depth Image Estimation Method Through Smoke

According to the double-Gamma distribution model, when there is no smoke obstruction, the echo signal is mainly composed of the reflected photons from the target. The echo signal approximately conforms to the Gaussian distribution as represented by Formula (A2). When there is smoke obstruction, the echo signal is obtained by superimposing two Gamma distribution signals. The echo signal will deviate from the Gaussian distribution of Formula (A2). Therefore, we plan to determine whether there is smoke interference by calculating the similarity between the echo signal and the Gaussian distribution.

Here, we introduce an instrument response function (IRF) to represent the Gaussian distribution of the target echo signal. The time-domain distribution of the IRF is mainly influenced by factors such as the laser pulse width, the time jitter of the detector, and the time jitter of the electronic timing system. The IRF is usually obtained through long-term tests on the target under indoor conditions. By using the measured IRF for similarity calculation, the reliability of smoke obstruction interference discrimination can be improved.

**Figure 1:**
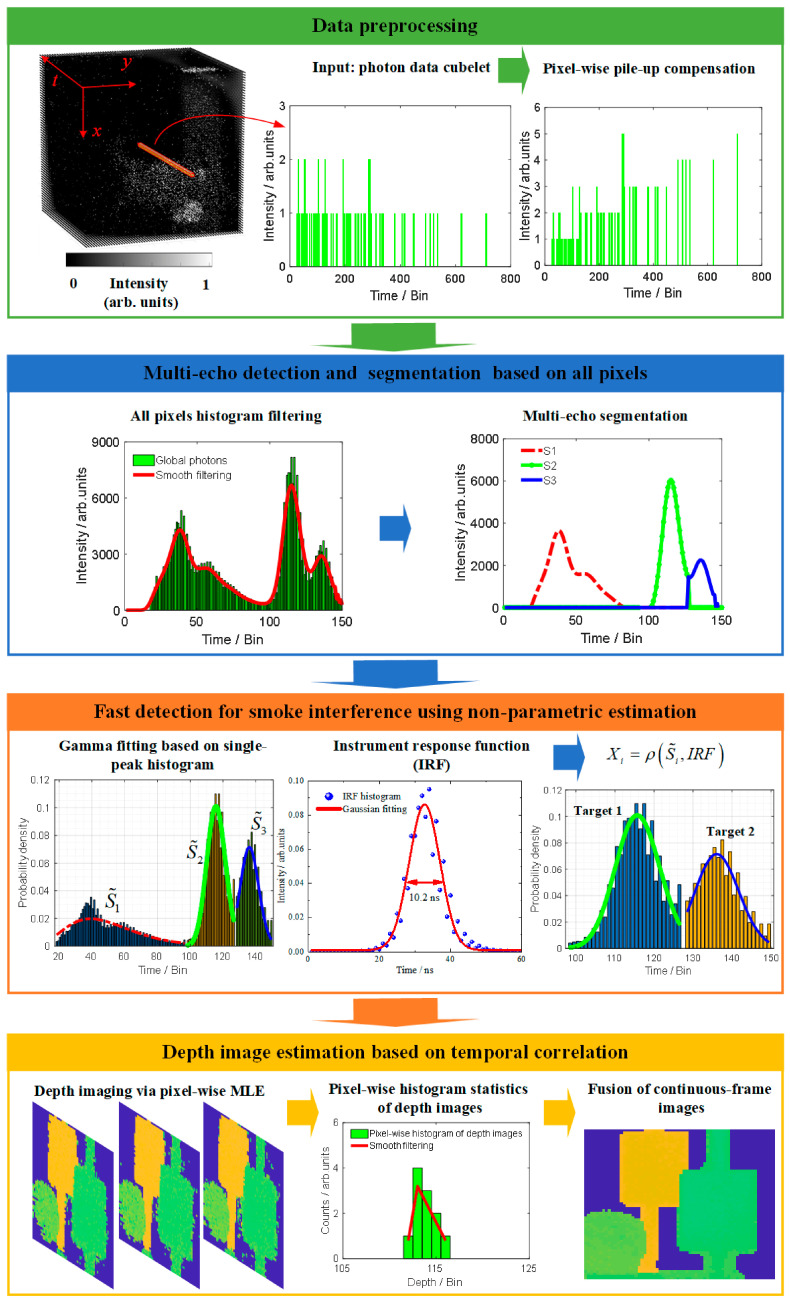
The algorithm flow for depth imaging through smoke based on non-parametric estimation.

The non-parametric rapid smoke detection and depth imaging algorithm for the array Gm-APD LiDAR proposed in this paper is shown in Figure 1. The processing steps of the algorithm are as follows.

1. **Data preprocessing:** The input echo signal histogram is pile-up compensated by pixel-wise, and then the compensated array histogram data is superimposed to obtain the full-pixel echo signal histogram.
2. **Multi-echo detection and segmentation based on all pixels:** For the echo signals after the pile-up compensation and superposition, the kernel density estimation algorithm [23] is used for smoothing and fitting. Then, echo peak searching is employed to segment the multiple echo signals, resulting in the echo peak data set *S* = [*S*_1_, *S*_2_, …, *S*_n_] and the data segmentation point set *t* = {[*t*_1_], [*t*_2_], …, [*t_n_*]}.
3. **Fast detection for smoke interference using non-parametric estimation:** For each segmented echo peak histogram data, perform Gamma fitting to obtain the fitting curve $\tilde{S}=\left[{\tilde{S}}_{1},{\tilde{S}}_{2},\ldots {\tilde{S}}_{n}\right]$. The Pearson correlation coefficient (PCC) was used to calculate the correlation coefficients between each fitting curve and the IRF. The PCC is adopted to quantitatively describe the degree of linear correlation between two discrete signal sequences. As one of the most commonly used statistical metrics for one-dimensional signal similarity analysis, its value ranges from −1 to 1. For discrete sampled signals with finite length, the calculation formula of the PCC is presented as follows.
  (5) $$\rho (x,y)=\frac{\sum_{i=1}^{n}\left(x_{i}-\bar{x}\right)\left(y_{i}-\bar{y}\right)}{\sqrt{\sum_{i=1}^{n}{\left(x_{i}-\bar{x}\right)}^{2}}\sqrt{\sum_{i=1}^{n}{\left(y_{i}-\bar{y}\right)}^{2}}}$$
  where *x* and *y* denote two discrete signal sequences. $\bar{x}$ and $\bar{y}$ represent the sample mean of the corresponding signal sequences. Formula (6) is adopted to fit the curve set $\tilde{S}=\left[{\tilde{S}}_{1},{\tilde{S}}_{2},\ldots {\tilde{S}}_{n}\right]$, and the correlation coefficient set *X* = [*X*_1_, *X*_2_, …, *X_n_*] is thereby obtained. If the correlation coefficient *X_i_* is less than or equal to the correlation coefficient threshold *X_thor_*, this echo peak is determined to be a smoke signal. At this time, the data segmentation point is *t_i_*, and the number of photons within the *t_i_* range in the echo data of each pixel of the array Gm-APD LiDAR is eliminated. If the correlation coefficient *X_i_* is greater than the correlation coefficient threshold *X_thor_*, this peak is determined to be the target signal. There is no need to segment this part of the data. Different from traditional Pearson correlation-based detection methods that only achieve simple similarity judgment, our nonparametric estimation strategy further combines statistical distribution characteristics of echo photons and Gamma fitting features. It realizes joint interference discrimination and depth parameter estimation, which effectively breaks through the limitation of single correlation analysis and reflects the novelty of the proposed method.
  (6) $$X_{i}=\rho \left({\tilde{S}}_{i},IRF\right)$$
4. **Depth image estimation based on temporal correlation:** Based on the noise-removed array data, the depth image is reconstructed using the pixel-wise maximum likelihood estimation (MLE) algorithm. The adjacent reconstructed images have strong structural similarity within a short time interval. This is manifested as follows: In consecutively reconstructed multi-frame depth images, the correctly estimated target distance values at the same pixel points exhibit prominent spatial clustering, while incorrectly estimated noise values are randomly distributed with obvious discreteness. Accordingly, pixel-level histogram statistics are performed on the depth information of the reconstructed consecutive multi-frame depth images. The peak position of each histogram is adopted as the distance estimation for the corresponding pixel in the current frame. By integrating consecutive multi-frame information and conducting pixel-wise fusion of distance signals, the final fused depth image can be obtained.

### 2.3. Experimental Scene and System Description

**Figure 2:**
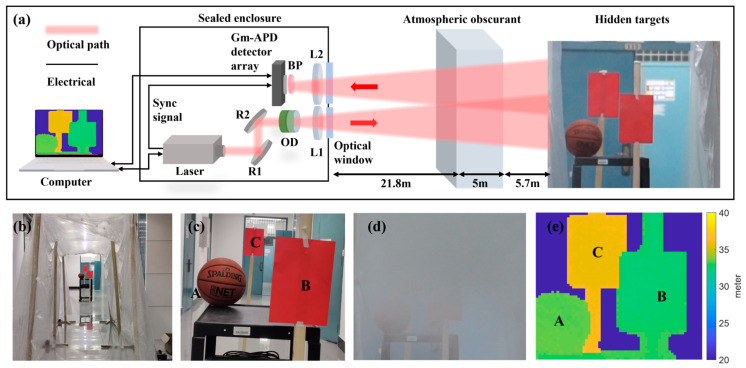
Schematic diagram of the structure and working principle of the array Gm-APD LiDAR. (**a**) The optical setup includes mirrors (R1 and R2), an optical diffuser (OD), lenses (L1 and L2), band pass filters (BP), and the Gm-APD detector array. (**b**) Scene diagram of smoke shelter and target position. (**c**) Experimental scene image without smoke obstruction. (**d**) Experimental scene image with smoke obstruction. (**e**) Depth image of the target without smoke obstruction, used as a reference image for evaluating the algorithm’s performance.

To verify the depth imaging performance of the proposed algorithm in actual smoke environments, an indoor experimental platform for depth imaging under artificial smoke occlusion interference is established in this paper. Based on the experimental data collected in the indoor artificial smoke environment, the performance of the proposed algorithm is validated. The experimental scenario is shown in Figure 2, which contains three targets: Target A (basketball) is 33.2 m away from the LiDAR, Target B is 32.5 m away, and Target C is 35.6 m away. A 5 m-long smoke shielding area is arranged on the optical path between the LiDAR and the targets, and the front end of this shielding area is 21.8 m from the LiDAR.

The array Gm-APD LiDAR used in the experiment has a resolution of 64 × 64 pixels. The central laser wavelength is 1064 nm, and the laser pulse width is 10 ns. Each pixel unit is integrated with an independent time-to-digital converter (TDC) module. The time bin resolution is 1 ns, which corresponds to a distance measurement resolution of 15 cm. The core system parameters are summarized in Table 1.

**Figure 3:**
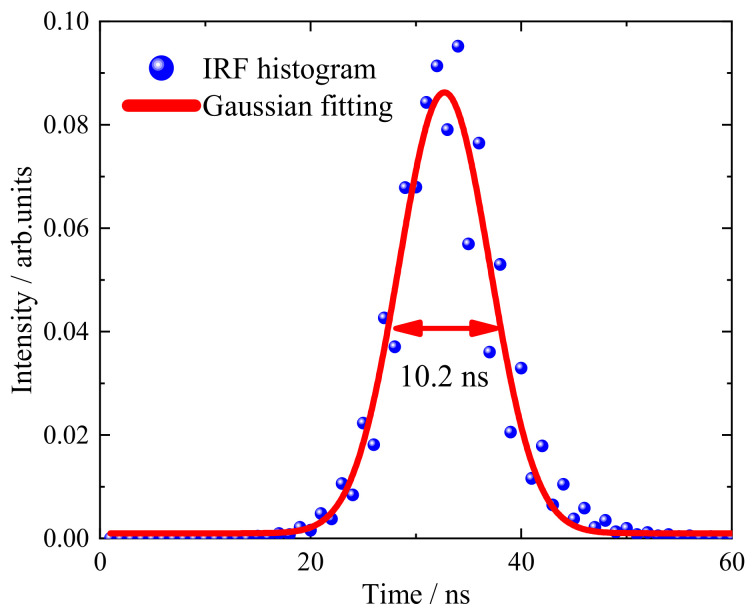
Instrument response function test histogram and Gaussian fitting results.

In order to calculate the Pearson correlation coefficient between the Gamma fitted curve of the echo data histogram and the IRF, we first need to test the IRF. When there is no smoke obstruction, the system parameters of the LiDAR are adjusted to make the depth image of target B complete and clear. Then, long-term data collection is carried out on target B. To ensure the accuracy of the IRF test, 20 × 20 pixels of echo signals within the image area of target B are selected for summation. The average intensity of the single-pulse echo signal is calculated by averaging, and the result is shown in Figure 3. Using Formula (A2) for fitting, the FWHM *τ_p_* was determined to be 10.2 ns. Subsequently, the Gaussian-fitted curve is used as the IRF to calculate the correlation coefficient.

### 2.4. Evaluation Criteria and Comparison Algorithms

In this study, two indicators, namely the occlusion rate (*OR*) and the average attenuation length (*N_AL_*), are used to describe the interference strength of atmospheric obscurants. The occlusion rate is calculated as follows:

(7) $$OR=\frac{m}{M}\%$$

where *m* denotes the number of pixels blocked by the atmospheric obscurant, and *M* denotes the total number of pixels, with *M* = 4096. The average attenuation length *N_AL_* is calculated according to the attenuation length formula [7], as follows:

(8) $$N_{AL}=\alpha l=-\frac{1}{2}\ln\left(\sum_{i=1}^{m}\frac{n(i)}{N(i)}\right)$$

where ln denotes the natural logarithm, *n* is the number of returned photons in the presence of the obscurant, and *N* is the number of returned photons measured without the obscurant. *l* represents the one-way propagation distance in the obscurant, and *α* denotes the attenuation coefficient of the obscurant.

To assess the reconstructed images, we use two metrics: target recovery (TR) and root mean square error (RMSE). The target recovery is defined as follows:

(9) $$\mathrm{TR}=\frac{\sum_{i=1}^{m}m_{ef}(i)}{m}$$

(10) $$m_{ef}(i)=\left\{\begin{array}{c} 1\\ 0 \end{array}\begin{array}{c} ,\\ , \end{array}\right.\begin{array}{c} \left|d(i)-d_{s}(i)\right|\leq d_{th}\\ \left|d(i)-d_{s}(i)\right|>d_{th} \end{array}$$

where *d* represents the reconstructed distance, *d_s_* is the ideal distance, *dth* is the distance error threshold, and *m_ef_* denotes the number of pixels whose distance errors fall within the threshold range. The root mean square error (RMSE) is defined as follows:

(11) $$\mathrm{RMSE}=\sqrt{\frac{\sum_{i=1}^{M}{\left[d(i)-d_{s}(i)\right]}^{2}}{M}}$$

A TR value closer to 1 indicates better target recovery performance. A smaller RMSE indicates higher accuracy in the reconstructed target depth image. The following methods are selected for comparison.

1. Peak selection algorithm (PSA) [32]: this classical method estimates the target distance by searching for the detection peak in the observation histogram. The target bin is identified as the one with the maximum number of firings, while the non-target bins contain fewer counts.
2. Time gating algorithm: this method removes smoke noise photons by segmenting photons according to their flight time. Since smoke is usually located in front of the target, the backscattered photons from smoke return earlier than the photons reflected from the target.
3. All parameter estimation algorithm (APEA) [23]: this probabilistic method estimates pixel-wise fog parameters directly from the measured data without calibration or prior information. It helps distinguish fog-reflected background photons from signal photons reflected by the occluded target.

## 3. Results

### 3.1. Correlation Coefficient-Based Smoke Signal Identification Results

**Figure 4:**
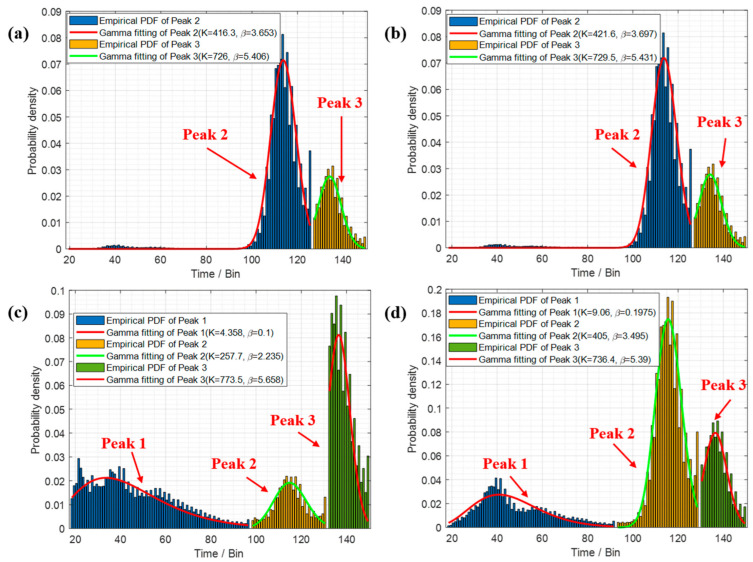
The histogram distribution of the echo signals after all-pixel superposition and the results of multi-peak fitting. (**a**,**b**) show the histograms and Gamma fitting results of targets A, B, and C when there is no smoke obstruction. (**c**,**d**) show the histograms and Gamma fitting results of smoke and targets A, B, and C when there is smoke obstruction.

The dynamic smoke occlusion experiment in this paper is a data collection experiment that lasts for 168 consecutive seconds. At the same time, a data collection experiment lasting for 168 consecutive seconds was also conducted without smoke occlusion. At the 60th second and the 120th second, the histogram statistics of the echo signals are conducted for the 250 frames of data in both smoke-free and smoke-impeded conditions. As shown in Figure 4, the horizontal axis denotes the detector time bin value, where one time bin corresponds to 1 ns. The vertical axis represents the normalized probability density, which is a dimensionless parameter. Notably, the probability density magnitudes of different signal peaks are independent of one another, and such values cannot reflect the actual intensity of the echo signal histogram. The histogram corresponds to the segmented target peaks, and the solid lines represent the Gamma fitting results of each segmented peak.

Figure 4a,b show the histograms and their fitted curves without smoke obstruction at 60 s and 120 s respectively. Without smoke obstruction, the histograms of the reference image targets A, B, and C in Figure 2e exhibit a bimodal distribution. Since the distance between targets A and B is 0.7 m, the corresponding photon one-way flight time difference is 4.7 ns, which is less than 1/2 of *τ_p_* (5.1 ns) fitted in Figure 3. Therefore, the echo signals of targets A and B overlap significantly in the time domain. In Figure 4a,b, Peak 2 represents the histograms of targets A and B, while Peak 3 represents the histogram of target C. Figure 4c,d show the histograms and their fitted curves during the periods of smoke obstruction at 60 s and 120 s respectively. Peak 1 represents the backscattering histogram of the smoke, Peak 2 represents the histograms of targets A and B, and Peak 3 represents the histogram of target C. It can be observed that whether it is the target peaks or the smoke echo peaks, the Gamma distribution model can fit the histograms quite well.

**Figure 5:**
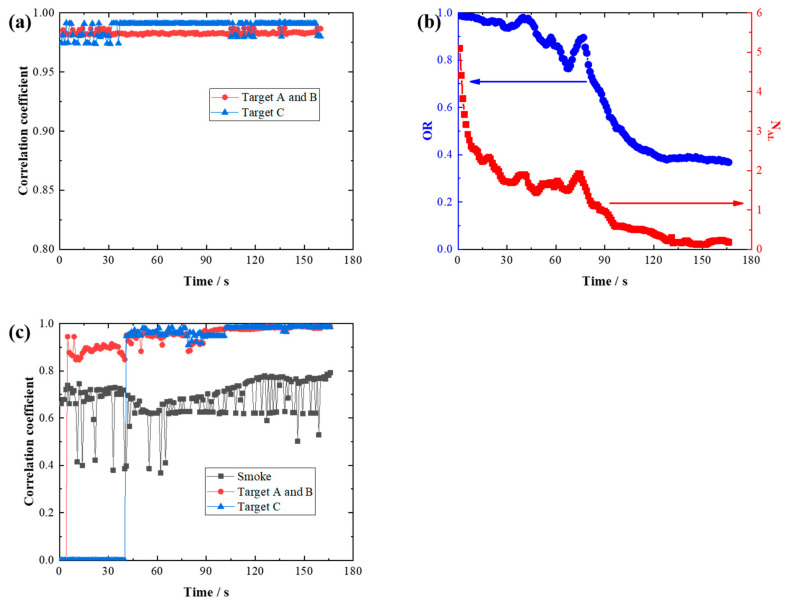
The calculation results of the correlation coefficients of the echo signals under continuous testing conditions. (**a**) When there is no smoke obstruction, the calculation results of the correlation coefficients of the echo signals of targets A and B, and target C. (**b**) When there is smoke obstruction, the obstruction rate and the distribution of average attenuation length calculated at different times. (**c**) When there is smoke obstruction, the calculation results of the correlation coefficients of the echo signals of smoke, targets A and B, and targets C.

To achieve the calculation of correlation coefficients under different conditions, this paper divides the 168 s array data into intervals of 1 s. For each time interval, the first 250 frames of data are selected for histogram statistics, which is used to calculate the correlation coefficient. The results are shown in Figure 5. The horizontal axis in the figure represents time interval in seconds. The vertical axes in Figure 5a,c denote the calculated correlation coefficients, which are dimensionless parameters. The vertical axis in Figure 5b stands for the occlusion rate (*OR*) and average attenuation length (*N_AL_*), both of which are dimensionless parameters.

Figure 5a presents the distribution of the correlation coefficient calculation results for targets A and B (corresponding to Peak 2 in Figure 4) and target C (corresponding to Peak 3 in Figure 4) when there is no smoke obstruction. Figure 5b shows the test results of the smoke obscuration rate and the average attenuation length at different times when smoke is present. The left vertical axis represents the target obscuration rate (in blue, as a scatter plot), and the right vertical axis represents the average attenuation length (in red, as a scatter plot). During the continuous test period from the 1st second to the 168th second, the occlusion rate decreases from the maximum of 98% to 36%, and the average attenuation length decreases from 5.1 to 0.2.

Figure 5c shows the distribution of the correlation coefficient calculation results for the smoke-obscured smoke (corresponding to Peak 1 in Figure 4), target A B (corresponding to Peak 2 in Figure 4), and target C (corresponding to Peak 3 in Figure 4). Due to the relatively large average attenuation length and occlusion rate of the smoke, in the first 4 s of the test, Peak 2 and Peak 3 are submerged in noise, and at this time the correlation coefficients of the two peaks are both zero. In the first 40 s of the test, Peak 3 is submerged in noise, and at this time the correlation coefficient of this signal peak is zero. Excluding the cases where the correlation coefficient is zero, the correlation coefficients of Peak 2 and Peak 3 are both greater than 0.83. The mean correlation coefficients are 0.9483 ± 0.0016 and 0.9701 ± 0.0003, respectively. Compared with the correlation coefficients without smoke obstruction, they have decreased by 0.0347 and 0.0178 respectively. The correlation coefficients of Peak 1 are all no greater than 0.8, and the average correlation coefficient is 0.6718 ± 0.0078. Compared with the correlation coefficients of Peak 2 and Peak 3 without smoke obstruction, they have decreased by 0.3112 and 0.3161 respectively. Although the obstruction of smoke would result in the correlation coefficient of the target signal peak being less than the result without smoke obstruction, it is still much smaller than the reduction in the correlation coefficient of the backscattered signal due to smoke (the difference is approximately 0.28).

To test whether the correlation coefficient can be used as a non-parametric feature for the discrimination of smoke obstruction interference, the depth images reconstructed from different data frames were used for verification. As shown in Figure 5c, the correlation coefficients of Peak 2 and Peak 3 are both higher than 0.83, while all correlation coefficients of Peak 1 are no greater than 0.8. Therefore, we set the correlation coefficient threshold between 0.8 and 0.83. Here, the correlation coefficient threshold *X_thor_* is set at 0.82. If the correlation coefficient calculated from a single depth image data is less than the *X_thor_*, it is determined that there is smoke obstruction. In this paper, the 168 s of smoke-obstructed data and the 168 s of non-smoke-obstructed data are respectively divided into 336 groups at a 1 s interval. During each test, 100 groups of smoke-obstructed data and 100 groups of non-smoke-obstructed data are randomly selected respectively. The number of data frames (F) for depth image reconstruction is set to 100, 250, 500, 1000, and 1500 (with a total of 2500 data frames in each group). The discrimination results based on the correlation coefficient are shown in Table 2.

### 3.2. Depth Imaging Results Based on Non-Parametric Estimation

**Figure 6:**
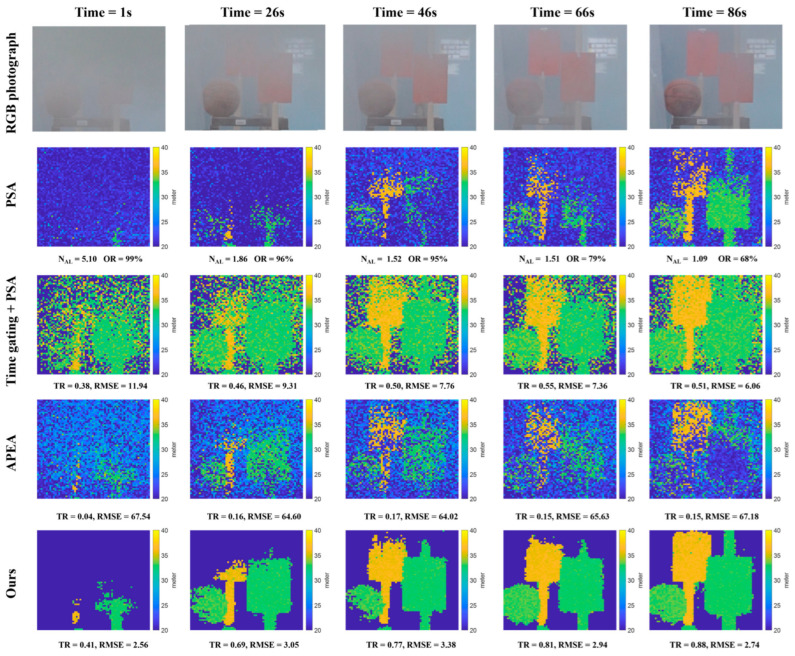
Comparison of the results of reconstructing depth images at different times.

To verify the performance of the proposed algorithm for depth imaging through smoke, this paper reconstructs the depth images based on the continuously collected data. The reconstruction results are shown in Figure 6. The number of data frames required for reconstructing each depth image is 250. The first row shows the real-time RGB image of the smoke experiment scene. The second row presents the depth images reconstructed by the PSA algorithm. Due to the obstruction caused by the smoke, the PSA algorithm has significant errors in estimating the depth image. There are a large number of smoke-impeded pixels in the reconstructed depth image. The depth images are similar to the RGB images of the scene. For this purpose, we used the depth images reconstructed by the PSA algorithm to calculate the occlusion rate and the average attenuation length.

The average attenuation lengths corresponding to the reconstructed data at 1 s, 26 s, 46 s, 66 s and 86 s are 5.10, 1.86, 1.52, 1.51 and 1.09 respectively, and the occlusion rates are 99%, 96%, 95%, 79% and 68% respectively. As shown in the third row of Figure 6, the result of depth image reconstruction combining Time gating and PSA is presented. The depth images contain the contour information of the target, but there are many noise pixels in the images. The fourth row in Figure 6 shows the depth images reconstructed by the APEA. The APEA reconstructs a small number of target pixels at different times. Among the three algorithms, the APEA has the smallest target TR value and the largest RMSE value. It is worth noting that the RMSE values in Figure 6 are expressed in the number of time bins. A time bin represents the minimum time interval of the detector, which is determined by the device hardware. For the array Gm-APD lidar adopted in this work, the time bin is 1 ns, corresponding to a photon one-way flight distance of 0.15 m. The last row presents the depth images reconstructed by the algorithm proposed in this paper. Our algorithm not only can adaptively distinguish the interference caused by smoke, but also has a high depth-imaging capability.

**Figure 7:**
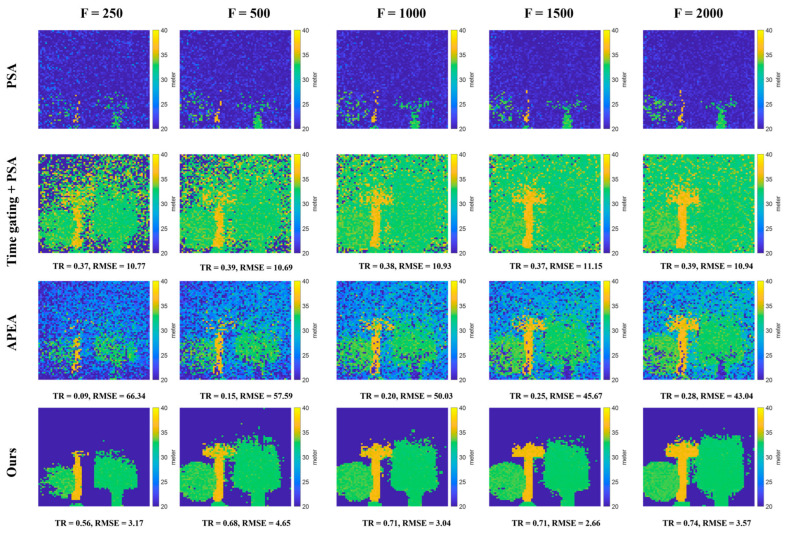
When the occlusion rate is 96% and the average attenuation length is 2.29, the comparison results of depth images reconstructed from different data frame numbers.

To test the depth-imaging capability of the algorithms under different numbers of data frames, this paper conducts depth imaging on a dynamic smoke scene with an occlusion rate of 96% and an average attenuation length of 2.29. The result of depth image reconstruction is shown in Figure 7. The number of data frames (F) required for depth image reconstruction is 250, 500, 1000, 1500 and 2000 respectively. Under different reconstruction frame numbers, the depth images reconstructed by the algorithm proposed in this paper have the highest TR value and the smallest RMSE value. As the number of data frames increases, the TR value gradually rises. When the number of data frames is 1000, the TR value and RMSE value of the proposed algorithm are 0.71 and 3.04 respectively. Compared to when the number of data frames is 2000, the TR value decreased by only 0.03, while the RMSE value increased by 0.53. When the number of data frames is 1000, the TR value of the depth image reconstructed by the Time gating and PSA is 0.38, and the RMSE value is 10.93.

**Figure 8:**
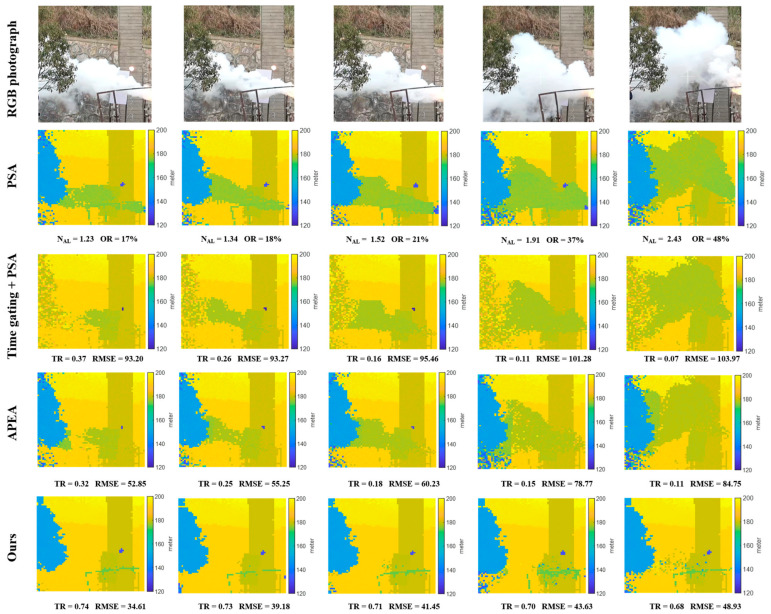
Comparison of interference suppression depth-imaging results in outdoor dynamic smoke occlusion environment.

The proposed algorithm is applied to the outdoor scenario with dynamic smoke occlusion and interference. The corresponding depth-imaging results are presented in Figure 8. The five columns of data represent continuous data acquisition from left to right, with a time interval ranging from 3 to 9 s. The first row shows the real-time RGB image of the smoke occlusion scene. The second row shows the depth image reconstructed by the PSA using 1000 frames of data. The calculation results are displayed below the reconstructed depth images of the PSA. During the dynamic process from left to right, the obscuration rate *OR* of the smoke increased from 17% to 48%, and the average attenuation length *N_AL_* increased from 1.23 to 2.43. These calculation results are consistent with the changes in the RGB smoke occlusion of the scene. The results demonstrate that, in the outdoor scenario with dynamic smoke occlusion interference, the depth images reconstructed by the algorithm proposed in this paper achieve the highest TR and the lowest RMSE.

## 4. Discussion

### 4.1. Discrimination of Smoke Interference Signals Based on Correlation Coefficient Calculation

During the continuous test period from 1 s to 168 s, the smoke occlusion rate varied from 98% to 36%, while the average attenuation length changed from 5.1 to 0.2. Under smoke-free conditions, the correlation coefficients between the target echo signals and the instrument response function (IRF) were consistently close to 1, indicating high similarity and good stability of the target returns. Specifically, the mean correlation coefficient of Peak 2 was 0.983016 ± 0.000002, and that of Peak 3 was 0.987905 ± 0.00003.

When smoke occlusion was present, the correlation coefficients of the target echo peaks decreased slightly compared with those obtained without smoke interference. However, this decrease was still much smaller than that observed for smoke backscattering signals. In particular, the reduction in the correlation coefficient for smoke backscatter was approximately 0.28 larger than that for the target echoes. This distinct difference indicates that the correlation coefficient can effectively separate target returns from smoke interference at the signal level without relying on explicit echo model fitting. This finding highlights the key distinction from conventional model parameter estimation algorithms [23,24,25,26,27,28], in that rapid discrimination of smoke occlusion can be achieved directly at the signal level.

The experimental results further show that, in the absence of smoke occlusion, the discrimination accuracy reached 100% using 100 reconstructed frames. In the presence of smoke occlusion, 100% smoke detection accuracy was achieved using 250 reconstructed frames. These results demonstrate that the correlation coefficient can serve as a robust non-parametric feature for determining whether a depth image is affected by smoke interference. This capability is particularly important in dynamic smoke environments, where rapid and reliable discrimination is required before subsequent depth reconstruction.

### 4.2. Comparison of Depth Imaging Results Based on Non-Parametric Estimation

Compared with traditional time-of-flight isolation and model-based estimation algorithms [23], the proposed method achieves the highest target recovery (TR) and the lowest root mean square error (RMSE) under both indoor and outdoor smoke environments. For example, when the average attenuation length is 1.52 and the occlusion rate reaches 95%, the proposed method restores 77% of the target pixels, while the RMSE is only 3.38 bins, corresponding to approximately 0.51 m. Compared with the Time gating and PSA, the mean TR at five representative moments increases by at least 23.2%, while the mean RMSE decreases by at least 65.4%. Compared to the images reconstructed by APEA, these two indicators have improved more significantly.

In comparison with the Time gating + PSA method, the proposed algorithm reconstructs the depth image from 1000 frames with an 86.8% improvement in TR and a 72.2% reduction in RMSE. In addition, the proposed method requires fewer data frames to achieve an image quality comparable to that obtained by reconstruction using approximately twice the number of frames. This result indicates that the proposed method has a clear advantage in data efficiency, which is beneficial for high-speed 3D imaging in practical smoke-obscured environments. Compared with the typical pixel-wise model parameter estimation algorithm (APEA), the proposed method consistently yields the highest TR and the lowest RMSE under different interference conditions. These results indicate that the proposed algorithm provides stronger anti-interference capability and better depth-reconstruction robustness than conventional model-based approaches.

From the perspective of the state of the art, many previous studies on imaging through atmospheric obscurants have mainly relied on parametric signal modeling or iterative reconstruction. For example, Satat [23] modeled fog echoes and target echoes using Gamma and Gaussian distributions, respectively. Based on similar modeling ideas, Liu [24] proposed a single-parameter fog-removal method, while Peng [25] combined Gamma modeling with density-clustering-guided Gaussian fitting. Mau [26,27] further described smoke-contaminated echoes using a mixed log-normal and Gaussian model. Although these methods have demonstrated effectiveness in certain obscured environments, they generally depend on explicit model assumptions and pixel-wise parameter estimation, which increase computational complexity and reduce adaptability in dynamic smoke conditions.

By contrast, the proposed method does not require parametric estimation of the echo model. Instead, it identifies smoke occlusion by calculating the Pearson correlation coefficient between the superposed array echo signal and the IRF, and then performs fast denoising and multi-frame depth fusion. This non-parametric strategy avoids the complex pixel-wise fitting process required by conventional approaches and is therefore better suited to dense and dynamic smoke environments, where the occlusion state changes rapidly in both space and time.

In addition, several recent studies have improved imaging performance in scattering environments through other strategies, such as iterative reconstruction [20], dual-branch target tracking under water mist interference [21], and vortex coherence filtering in dense fog [22]. These works confirm the importance of advanced optical or computational methods for imaging through obscurants. However, few studies have specifically addressed rapid smoke interference discrimination and depth reconstruction for array Gm-APD LiDAR under dynamic smoke occlusion. In this respect, the present work extends the existing literature by providing a practical non-parametric framework that integrates single-frame smoke discrimination with efficient depth image reconstruction.

Overall, the results show that, in both indoor and outdoor smoke environments, the proposed method consistently achieves the highest TR and the lowest RMSE. This demonstrates that the proposed algorithm outperforms the conventional model parameter estimation algorithm (APEA) in terms of reconstruction accuracy, robustness, and adaptability to dynamic smoke interference. Therefore, the proposed method has strong practical potential for high-speed depth imaging in harsh atmospheric environments with severe smoke obscuration.

## 5. Conclusions

To sum up, this study proposes a non-parametric estimation for rapid smoke detection and a depth imaging algorithm suitable for array Gm-APD LiDAR. This algorithm does not require model parameter estimation of the echo signal. It can achieve rapid discrimination of smoke occlusion by calculating the Pearson correlation coefficient between the summed echo signals of all pixels of the array Gm-APD LiDAR and the instrument response function. In a dynamic smoke occlusion environment with an average attenuation length not exceeding 5.1, a 100% accuracy rate for smoke occlusion interference discrimination is achieved. The number of data frames is only 250. Based on the recognition results, efficient isolation of the noise photons from the backscattering of smoke is carried out for the echo data. Meanwhile, by combining the time correlation of the reconstructed depth images, pixel-level information fusion is performed on the continuously reconstructed depth images, thereby improving the target restoration degree of the reconstructed depth images. When the smoke obscuration rate is 96% and the average attenuation length is 2.29, depth imaging with a target restoration degree of 0.71 is achieved, which is an improvement of 86.8% compared to traditional algorithms. Compared with the model parameter estimation algorithm, the proposed algorithm has stronger anti-interference ability in both indoor and outdoor dynamic smoke environments. This research holds significant application value for the engineering application of the array Gm-APD LiDAR in the rapid depth imaging under complex atmospheric obstruction environments.

## Figures and Tables

**Table 1:** Summary of main system parameters.

System Parameter	Value
Laser wavelength/nm	1064
Laser pulse width/ns	10
Diameter of camera lens/mm	70
Divergence angle of laser/mrad	17.5
Divergence angle of camera lens/mrad	17.5
BP/nm	1064 ± 1
InGaAs Gm-APD camera	64 × 64 pixels
Photon detection efficiency (PDE)	25% at 1064 nm
Working wavelength/nm	980~1150
Timing jitter (camera)/ns	≤1
Dark count rate/kHz	10
Death time/μs	40 (single-hit model)
Bin width/ns	1
Histogram length	4000 Bins

**Table 2:** The identification results of smoke occlusion interference based on the correlation coefficient.

F	Total Number of Images Without Smoke (M)	Total Number of Images with Smoke (N)	Total Number of Smoke-Free Images Detections (m)	Total Number of Smoke Images Detections (n)	Accuracy Rate P (P = m/M or P = n/N)
100	100	0	100	0	100%
100	0	100	4	96	96%
250	100	0	100	0	100%
250	0	100	0	100	100%
500	100	0	100	0	100%
500	0	100	0	100	100%
1000	100	0	100	0	100%
1000	0	100	0	100	100%
1500	100	0	100	0	100%
1500	0	100	0	100	100%

## Data Availability

The data presented in this study are available upon request from the corresponding author.

## Abbreviations

The following abbreviations are used in this manuscript:

FWHM	Full width at half maximum
IRF	Instrument response function
PCC	Pearson correlation coefficient
MLE	Maximum likelihood estimation
OR	Occlusion rate
N_AL_	Average attenuation length
TR	Target recovery
RMSE	Root mean square error
PSA	Peak selection algorithm
APEA	All parameter estimation algorithm

## Appendix A

Because the Gm-APD detector measures time in discrete bins, the time variable *t* in the echo acquisition process is generally expressed by bin numbers. Here, *i* represents the *i*-th time bin. To mitigate the pile-up effect induced by dense atmospheric obscurants in array Gm-APD LiDAR, we model the observed histogram with a multinomial distribution. Accordingly, the probability *P*(*H*∣*S*) of observing histogram *H* for a single pixel under atmospheric obscurant conditions is written as follows:

(A1) $$\begin{array}{c} P\left(H|S\right)=\frac{N!}{H_{1}!H_{2}!\ldots H_{T}!\left(N-I^{T}H\right)!}P{\left(\mathrm{no}\mathrm{detections}\right)}^{N-I^{T}H}\prod_{i=1}^{T}P{\left(i\left|S\right.\right)}^{H_{i}}\\ \;\;\;=\frac{N!}{H_{1}!H_{2}!\ldots H_{T}!\left(N-I^{T}H\right)!}\exp{\left(-I^{T}S\right)}^{N-I^{T}H}\prod_{i=1}^{T}{\left\{\exp\left(-\sum_{k=1}^{i-1}S_{k}\right)-\exp\left(-\sum_{k=1}^{i}S_{k}\right)\right\}}^{H_{i}} \end{array}$$

where *T* represents the maximum time-bin index. ***I*** is a row vector of length *T* whose elements are all equal to 1. *S* denotes the corresponding column vector distribution.

## Appendix B

To extract target depth information, it is necessary to estimate the time-domain distribution of the target-reflected photons after converting the observed photon-count histogram into echo photon counts. The target echo signal *N_t_* can be described as the convolution of the transmitted laser pulse with the reflected signal intensity from target surfaces located at different depths. For modeling simplicity, a Gaussian function [33] is employed to characterize the target echo signal. The corresponding fitting model is given as follows.

(A2) $$N_{t}(t)=s\frac{2}{\tau_{p}}\sqrt{\frac{\ln2}{\pi }}\exp\left[\frac{-4\ln2{\left(t-t_{t}\right)}^{2}}{\tau_{p}^{2}}\right]$$

where *s* denotes the intensity coefficient of the reflected photons and is unitless. The full width at half maximum (FWHM) of the emitted laser pulse after reflection from the target surface is represented by *τ_p_*. Moreover, *t_t_* = 2*d*/*c*, where *d* denotes the target range and *c* is the speed of light.

When the emitted laser pulse travels through atmospheric obscurants, such as smoke, the multipath scattering caused by particles leads to pulse broadening in the backscattered smoke echo. Therefore, the Gamma distribution model [31] is adopted to describe the smoke backscattering echo signal. The corresponding expression for the smoke echo signal *N_f_* is given as follows.

(A3) $$N_{f}(t)=\delta_{f}\frac{{\beta_{f}}^{K_{f}+1}}{\Gamma (K_{f})}t^{K_{f}}\exp(-\beta_{f}t)$$

For the Gamma distribution model, if its shape parameter *K* is sufficiently large, the Gamma distribution approximates the Gaussian distribution. Therefore, we employ the double Gamma distribution model to represent the echo signal, and its specific form is as follows:

(A4) $$S(t)=\eta \delta_{f}\frac{{\beta_{f}}^{K_{f}+1}}{\Gamma (K_{f})}t^{K_{f}}\exp(-\beta_{f}t)+\eta \delta_{t}\frac{\beta_{t}^{K_{t}+1}}{\Gamma (K_{t})}t^{K_{t}}\exp(-\beta_{t}t)+\eta N_{b}+N_{d}(t)$$
